# Construction of a gE-Deleted Pseudorabies Virus and Its Efficacy to the New-Emerging Variant PRV Challenge in the Form of Killed Vaccine

**DOI:** 10.1155/2015/684945

**Published:** 2015-09-17

**Authors:** Tongyan Wang, Yan Xiao, Qingyuan Yang, Yuzhou Wang, Zhe Sun, Chaoling Zhang, Shijun Yan, Juan Wang, Linghua Guo, He Yan, Zhiyu Gao, Lilin Wang, Xiangdong Li, Feifei Tan, Kegong Tian

**Affiliations:** ^1^National Research Center for Veterinary Medicine, Road Cuiwei, High-Tech District, Luoyang 471003, China; ^2^College of Animal Science and Veterinary Medicine, Henan Agricultural University, Zhengzhou, Henan 450002, China

## Abstract

The new-emerging PRV variants plague the vaccinated pigs and caused huge economic loss to local pig industry in China since 2011. The current commercial PRV vaccines cannot provide complete protection as the new-emerging PRV variants are antigenically different from the classical viruses. It is urgent to develop more safe and effective PRV vaccines based on the current circulating field isolates. In this study, a gE gene-deleted PRV based on the PRV HN1201, a representative PRV variant, was generated and the efficacy was tested on 3-week-old pigs in the form of killed vaccine. After fatal PRV HN1201 challenge, all vaccinated pigs survived without showing any clinical symptoms, but all unvaccinated pigs exhibited pseudorabies-specific respiratory and neurological signs with 100% mortality rate within 6 days after infection. The vaccinated pigs developed high level of gB and neutralizing antibodies after vaccination which may correlate to the protection provided by vaccine. Therefore, this gE gene-deleted PRV could be a promising vaccine candidate for the control of currently epidemic pseudorabies in China.

## 1. Introduction

The pseudorabies virus (PRV), also known as Aujeszky's disease virus, is a herpesvirus in family Herpesviridae, subfamily Alphaherpesvirinae, and genus* Varicellovirus*. PRV is able to infect nearly all mammals, including ruminants, carnivores, and rodents, yet swine have been confirmed to be the natural hosts and reservoir of this virus [1]. In pigs, PRV infection may lead to nervous signs and death in newborn pigs, respiratory disorders in fattening pigs, and reproductive failure in sows and remain latently infected in the central nervous system such as trigeminal ganglions following clinical recovery [2]. In the past decade, PRV has been eradicated in US and some European countries through gene-deleted modified-live vaccines accompanied by the application of ELISA serologic tests [3]. However, since 2011, the new-emerging PRV variants in China have occurred among the vaccinated pigs with apparent pseudorabies- (PR-) related clinical symptoms and spread rapidly to many provinces in China, which constitute a serious animal health threat and an economic burden to Chinese pig industry [4].

Virus genome analysis showed that all isolates of Chinese PRV variants belonged to a relatively independent cluster in the phylogenetic tree, which indicates the current circulating PRV variants are antigenically different from the classical ones [5]. The commercial available PRV vaccines cannot provide full protection to the infection of PRV variants as the vaccinated pigs still displayed typical PR signs such as high fever, depression, anorexia, and systemic neurological signs with high morbidity and mortality after infection [6]. Therefore, it is imperative to develop more efficient vaccines based on current field circulating PRV isolates to control the disease.

In this study, a gE gene-deleted PRV mutant was generated from the RPV variant HN1201, a virulent field strain isolated in 2011. The inactivated virus was then combined with mineral adjuvant MONTANIDE ISA 206 and tested on 3-week-old pigs for the efficacy study. Our results showed that all vaccinated pigs survived without exhibiting any clinical signs after ensuing virulent PRV HN1201 challenge as compared with unvaccinated pigs that were all dead after 6 days of postchallenge (dpc). These results indicate this gE-deleted PRV mutant could be a promising DIVA (differentiating infected from vaccinated animals) vaccine candidate to control the circulation of PRV variants in China.

## 2. Materials and Methods

### 2.1. Cells and Virus

The wild-type PRV variant HN1201 has been previously described [7]. Vero cells were grown in Dulbecco's Modified Eagle's Medium (Gibco, USA) supplemented with 10% fetal bovine serum (HyClone, USA) at 37°C with a humidified 5% CO_2_ atmosphere, while viral infections were performed in DMEM supplemented with 2% FBS. PK-15 cells were cultured with same medium and conditions and were used in plaque assay and serum-virus neutralizing test.

### 2.2. Generation of PRV HN1201ΔgE

**Figure 1:**
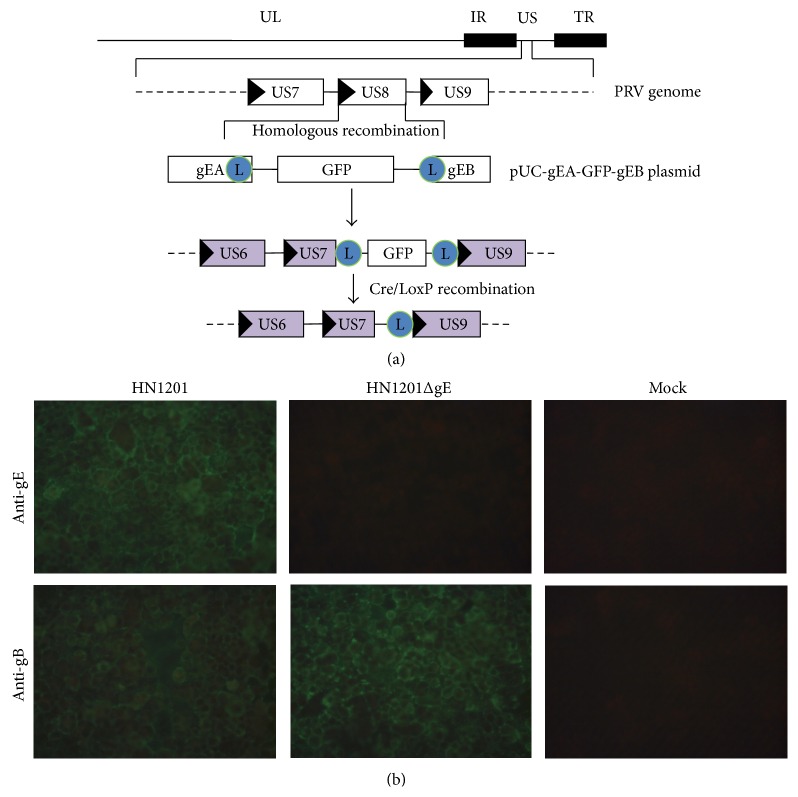
Schematic representation of the strategy used to produce infectious PRV HN1201ΔgE (a) and confirmation of PRV HN1201ΔgE-GFP by detection of gE and gB protein using indirect fluorescence assay (b).

To construct the transfer vector with homologous sequences to regions of the PRV genome, two pairs of primers (gEAF/R and gEBF/R) with appropriate restriction enzymes (*EcoR I/Xba I* and* Sph I/Hind III*) and loxP sequences (same orientation) were designed according to the sequence of PRV HN1201 for amplification of homologous arms gEA and gEB (Table 1). The gEA and gEB fragments were cloned into pUC19 vector (Invitrogen, USA) linearized with* EcoR I* and* Xba I* or* Sph I* and* Hind III* to obtain the transfer vector pUC-gEA-gEB. GFP expression cassette (under the control of CMV promoter and polyA terminator) was amplified with primers CMVU and SV40R (Table 1) and was cloned into the pUC-gEA-gEB to create pUC-gEA-GFP-gEB vector (Figure 1(a)).

PRV HN1201 genomic DNA was extracted by using the DNAzol Reagent (Invitrogen, USA) following the instructions of the manufacturer. Vero cells were seeded in six-well plates (6.0 × 10^5^ cells/well) and were cotransfected with pUC-gEA-GFP-gEB plasmid and PRV HN1201 genomic DNA using Lipofectamine 2000 reagent (Invitrogen, USA) according to the manufacturer's instructions. When a cytopathic effect (CPE) was observed, the culture supernatant was harvested and plated on the fresh Vero cells overlaid with medium containing 1% low-melting agarose. Based on the GFP expression, plaque purification was carried out to obtain the homogeneous viruses. The presence of the GFP gene and the absence of the gE gene were verified by PCR using GFP specific (CMVU and SV40R) and gE-specific (gE-DF/gE-DR) primers (Table 1). The expected gE-deleted virus expressing GFP was named as PRV HN1201ΔgE-GFP.

To remove the GFP gene cassette, the genomic DNA of PRV HN1201ΔgE-GFP was cotransfected with pBS 185 plasmid (expressing cyclization recombinant enzyme which can recognize loxP sequence and remove it from the genome) in Vero cells and the gE-deleted virus (PRV HN1201ΔgE) was obtained after purification by nonfluorescent plaques (Figure 1(a)).

### 2.3. Indirect Immunofluorescence Assay

Detection of gE or gB expression in PRV HN1201ΔgE or PRV HN1201-infected cells was performed by indirect immunofluorescence assay. PK-15 cells were inoculated at 1 MOI with each of the viruses for 24 h. Cells were fixed with cold acetone, permeabilized with 0.5% Triton X-100, and incubated with mouse anti-gE or anti-gB monoclonal antibody (Beijing TianTech Biotechnology, China) at 1 : 800 dilution for 2 h at 37°C. Cells were subsequently labeled with FITC-labeled goat anti-mouse IgG (Sigma-Aldrich, USA) at 1 : 400 dilution for 1 h at 37°C. The stained cell monolayer was visualized under fluorescence microscope (Olympus, Japan).

### 2.4. Virus One-Step Growth Kinetics and Plaque Size Determination

One-step growth kinetics was conducted to compare the growth kinetics of the PRV HN1201ΔgE with the parental virus PRV HN1201. PK-15 cell monolayer was infected with each virus at a MOI of 1. Cell supernatants were harvested at successive intervals after infection and stored at −80°C. The amount of infectious virus was determined by 50% tissue culture infectious dose (TCID_50_). Growth kinetics for each virus tested was performed in triplet, and the resulting titers were averaged.

Plaque sizes were determined at 48 hours by inoculating 500 TCID_50_ of virus on PK-15 cells. After 1-hour incubation with virus, the medium was aspirated and cells were overlaid with 1% low-melting point agarose containing 2% FBS in DMEM for plaque formation. For each virus, 100 plaques were randomly selected and their size was determined by ImageJ software (National Institutes of Health). Values were calculated in comparison to those of PRV HN1201 which was set at 100%. Average percentages and standard deviations were determined from three independent experiments.

### 2.5. Vaccine Preparation and Animal Experiment

The inactivated virus was prepared by incubating one part formalin (Sigma-Aldrich) with 1000 parts of PRV HN1201gE^−^ supernatant (10^8.67^ TCID_50_/mL) at 37°C for 48 hours. It was then homogenized with the mineral MONTANIDE ISA 206 adjuvant (SEPPIC, France).

Ten 3-week-old pigs free of PRV, porcine reproductive and respiratory syndrome virus (PRRSV), classical swine fever virus (CSFV), and porcine circovirus 2 (PCV2) were randomly divided into vaccinated group and unvaccinated group. Pigs in the vaccinated group were inoculated intramuscularly (i.m.) with 2 mL inactivated PRV HN1201ΔgE vaccine, and pigs in unvaccinated group received 2 mL DMEM medium. After vaccination, rectal temperature and clinical signs were recorded on a daily basis. The serum samples were collected to monitor PRV gB/gE and neutralizing antibodies at designated days. Four weeks after vaccination, all pigs were challenged intranasally (i.n.) with 1 mL (10^7.0^ TCID_50_/mL) virulent PRV variant HN1201. At 14 dpc, all surviving pigs were euthanized, and different tissue samples were collected for histopathology and immunohistochemistry examination. The animal trial in this study was approved by the Animal Care and Ethics Committee of China National Research Center for Veterinary Medicine and conventional animal welfare regulations and standards were also taken into account.

### 2.6. Blocking Enzyme-Linked Immunosorbent Assay (ELISA) and Serum-Virus Neutralizing Test (SNT)

PRV-specific gE (IDEXX, USA) and gB (BioChek, Holland) antibodies were detected by using commercially available ELISA kits according to the manufacturer's directions. Serum samples were tested by SNT for the PRV-specific neutralizing antibodies (NAbs). Briefly, serum samples were heat inactivated at 56°C for 30 min prior to performing the serum-neutralization assay. Two-fold serially diluted sera (50 *μ*L) were mixed with an equal volume 100 TCID_50_ of the PRV HN1201 in 96-well culture plates and incubated at 37°C for 1 h in 5% CO_2_ atmosphere. After incubation, 100 *μ*L of PK-15 cell suspension containing 2 × 10^4.0^ cells was added to each well. The inoculated cells were then incubated at 37°C for 5 days for development of CPE to determine the titers of PRV-specific NAbs, and the titers were expressed as the reciprocal of the highest dilution at which infection of the PK-15 cells was inhibited in 50% of the culture wells.

### 2.7. Histopathology and Immunohistochemistry Staining

The cerebellum, tonsil, lymph node, and lung samples were collected for hematoxylin and eosin (H&E) and immunohistochemistry staining. The H&E staining was operated automatically by Leica fully automatic dyeing machine according to standard procedures. The immunohistochemistry staining was performed as below. The prepared paraffin sections were mounted on APES-treated slides and incubated overnight at 37°C after 60°C 15-minute treatment. The slides were dewaxed as routine method by Leica automatic dyeing machine. The samples were blocked with 3% peroxide-methanol for 20 minutes at room temperature for endogenous peroxidase ablation and rinsed by Phosphate Buffer Solution (PBS) twice. All the following steps were carried out in a moisture chamber: (1) incubate with blocking buffer containing normal horse serum (Beijing Zhongshanjinqiao, China) with 1 : 20 dilution with PBS at 37°C for 20 minutes; (2) discard the horse serum and incubate in PRV monoclonal antibody 3B5 solution (Beijing Tian Tech Biotechnology, China) with 1 : 800 dilution in PBS (pH 7.3) at 37°C for half an hour and then 4°C overnight; (3) after rinsing with PBS three times, add HRP goat anti-mouse IgG (BTI, USA) with 1 : 100 dilution in PBS (pH 7.3) and incubate the slides for 1 hour at 37°C; (4) after rinsing with PBS three times, incubate the slides with AEC and keep them at room temperature without light for 5–10 minutes; (5) after rinsing with PBS three times, the slides are stained with hematoxylin (freshly prepared) 1 : 10 dilution for 10 seconds; (6) wash away the unbound hematoxylin by running water, and place the slides into water for 2 minutes; (7) dry the slides naturally and mount with water-soluble tablets seal (GVA) before visualizing by 200x microscope photographs.

### 2.8. Virus Shedding by PCR

To detect virus shedding after viral challenge, nasal swabs from vaccinated and unvaccinated pigs were collected daily and were subjected to PRV gE gene detection by PCR. Primers specific for gE gene were listed in Table 1. The number of pigs excreting virulent PRV after viral challenge was calculated and summarized in Table 2.

### 2.9. Statistical Analysis

Data was presented as mean ± SD. The differences in plaque areas of viruses, body temperature, and body weight gain of pigs between two groups were determined by using *t*-test in GraphPad Prism 5.0 Software (San Diego, CA). Differences were considered statistically significant when *P* < 0.05.

## 3. Results

### 3.1. Generation of PRV HN1201ΔgE

Two steps were performed to generate gE-deleted recombinant PRV. In the first step, a transfer plasmid pUC-gEA-GFP-gEB was constructed by inserting the gEA/gEB fragments and GFP expression cassette into pUC19 vector. Vero cells were cotransfected with pUC-gEA-GFP-gEB plasmid and genomic DNA of PRV HN1201 to generate gE-deleted/GFP-expressing virus PRV HN1201ΔgE-GFP after homologous recombination. In the second step, the genomic DNA of PRV HN1201ΔgE-GFP was extracted from GFP-positive cells and cotransfected with pBS185 CMV-Cre plasmid in Vero cells to remove GFP expression cassette by using Cre/LoxP recombinant system. The plaques without green fluorescence were screened and purified for six rounds, resulting in the final PRV HN1201ΔgE. PCR identification and sequencing results showed that gB gene, but not GFP and gE genes, was detectable from the genome of PRV HN1201ΔgE (data now shown). As shown by Figure 1(b), gB protein was detected by IFA in PK-15 cells infected with PRV HN1201 or PRV HN1201ΔgE, whereas the gE protein was only detected in PK-15 cells infected with PRV HN1201, which indicates the recombinant PRV HN1201ΔgE virus was successfully rescued.

### 3.2. Growth Properties of PRV HN1201ΔgE

**Figure 2:**
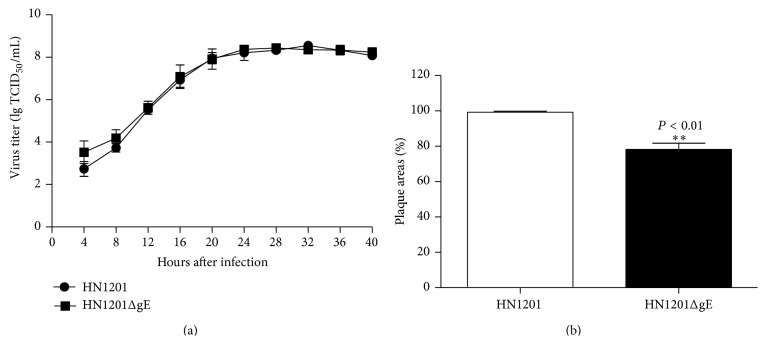
Replication kinetics (a) and plaque size (b) of PRV HN1201ΔgE and parental PRV HN1201. ∗∗ indicates *P* < 0.01.

To characterize the growth properties of PRV HN1201ΔgE, virus replication on PK-15 cells was analyzed by one-step growth kinetics and plaque assay. The growth feature of PRV HN1201ΔgE was virtually identical to that of parental PRV HN1201 in PK-15 cells as shown by Figure 2(a). However, the plaque areas of the reconstituted virus were significantly smaller than that formed by parental virus in PK-15 cells (Figure 2(b)).

### 3.3. Protection of Vaccinated Pigs from Virulent PRV HN1201 Challenge

#### 3.3.1. Clinical Symptoms after Viral Challenge

**Figure 3:**
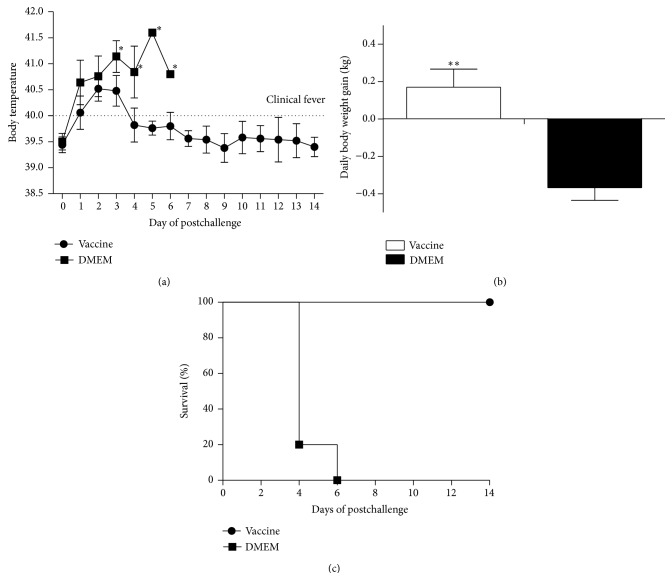
Body temperature (a), daily body weight gains within first 4 days (b), and survival rate (c) of pigs after PRV HN1201 challenge. ∗ indicates *P* < 0.05 and ∗∗ indicates *P* < 0.01.

PRV HN1201ΔgE was inactivated and combined with mineral MONTANIDE ISA 206 adjuvant for the pig vaccination. All pigs in both vaccinated group and control group remained clinically healthy and showed no adverse reactions after vaccination (data not shown). After 28 days, the pigs in both groups were challenged intranasally with 10^7.0^ TCID_50_ virulent PRV variant HN1201. The unvaccinated pigs developed high fever (range from 40.5°C to 41.7°C, Figure 3(a)) which started from 1 dpc and showed typical PR-clinical symptoms such as respiratory distress, excessive salvation, and neurological signs including convulsion, muscle tremor, posterior paralysis, and ataxia. Four pigs in unvaccinated group were found dead at 4 dpc and one pig was found dead at 6 dpc (Figure 3(c)). On the contrary, pigs in vaccinated group only showed a transient fever within four days after challenge and came back to normal body temperature after that. There were no other obvious clinical symptoms which were observed in the vaccinated group. As for the body weight gain in these two groups. The unvaccinated pigs kept losing body weight after PRV HN1201 challenge in the first 4 days of postchallenge (Figure 3(b)). By contrast, the vaccinated pigs gained the body weight with average of 0.2 kg during the same period.

As for the virus shedding, the virulent challenge virus could be detected in two vaccinated pigs at 2 dpc and no virus could be detected after 4 dpc (Table 2). By contrast, the challenged virus could be detected in two unvaccinated pigs at 1 dpc and all pigs after that until the death of unvaccinated pigs.

#### 3.3.2. Pathological Examination

**Figure 4:**
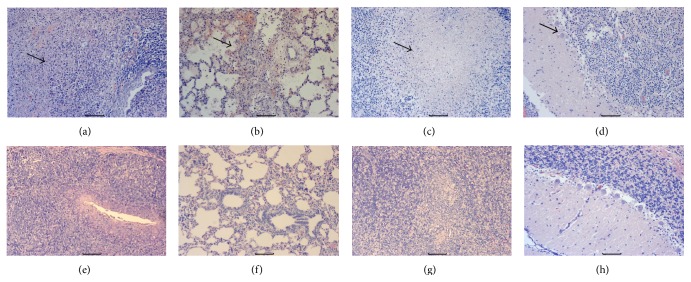
Histopathological results of unvaccinated (a–d) and vaccinated pigs (e–h). (a) Tonsil. Tonsillar lymphoid tissue necrosis and formation of big necrotic foci. (b) Lung. Massive vascular congestion. (c) Mesenteric lymph node. Vascular dilatation and formation of big necrotic foci. (d) Cerebellum. Purkinje cell degeneration and necrosis. (e–h) Tonsil, lung, lymph node, and cerebellum samples from vaccinated pigs. Original magnification ×200.

**Figure 5:**
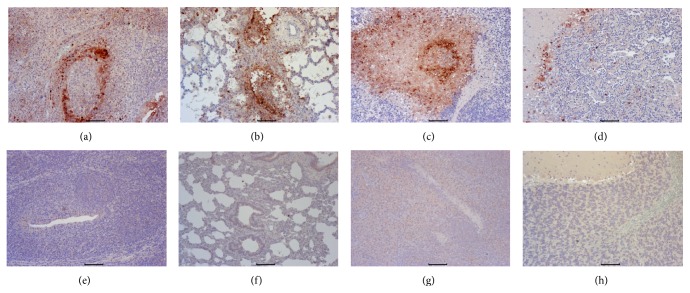
Immunochemistry staining of tonsil (a, e), lung (b, f), mesenteric lymph node (c, g), and cerebellum samples of unvaccinated (a–d) and vaccinated pigs (e–h). Original magnification ×200.

The euthanized pigs were subject to necropsy for pathological and histopathological examination. All unvaccinated pigs showed severe pulmonary consolidation and necrosis in the lung, encephalic hemorrhage in the cerebellum, and hemorrhage and necrosis in the tonsil and lymph nodes. There were no visible gross pathological changes for the pigs in the vaccinated group. Histopathological examination results showed that the unvaccinated piglets had hemorrhages and necrosis in tonsil, lung, and lymph node samples (Figures 4(a)–4(c)). The infected pigs also showed Purkinje cell degeneration and necrosis in the cerebellum (Figure 4(d)). None of vaccinated piglets displayed any histopathological changes. Consistent with pathological results, immunochemistry results also showed strong positive staining in the tonsil, lung, lymph node, and cerebellum samples of unvaccinated pigs (Figures 5(a)–5(d)). No positive staining was detected in the above tissues of vaccinated pigs (Figures 5(e)–5(h)).

#### 3.3.3. Antibody Response after Vaccination and Challenge

**Figure 6:**
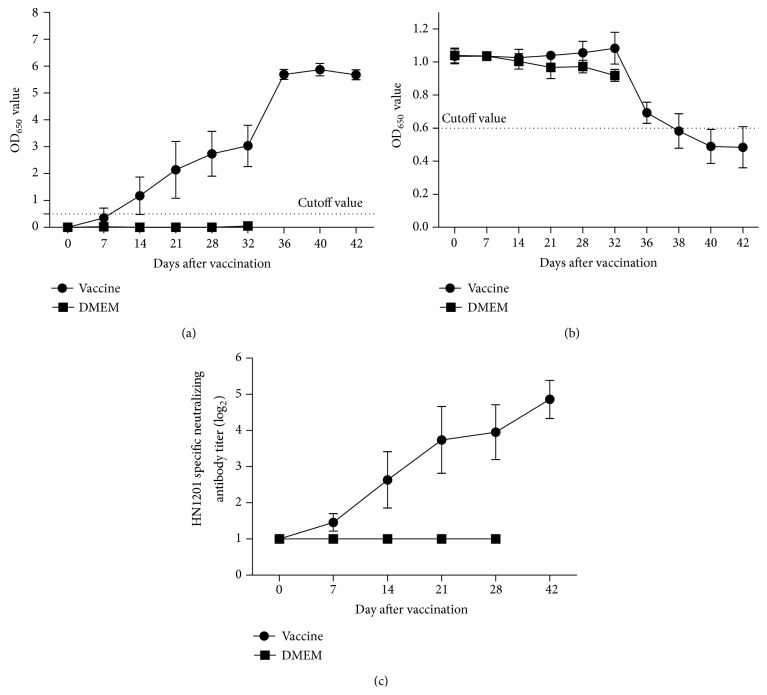
Profile of PRV gB-specific (a), gE-specific (b), and neutralizing antibody (c) responses after vaccination.

The gB-specific antibodies were detected in pigs immunized with PRV HN1201ΔgE at 7-day postimmunization (dpi), and all pigs seroconverted positively at 14 dpi (Figure 6(a)). Antibody titers kept steadily increasing with average 2.7 of OD_650_ after immunization before jumping sharply to more than 5 of OD_650_. By contrast, the unvaccinated pigs did not develop any gB antibodies after inoculating with DMEM medium and all pigs were dead before any gB antibodies could be detected. As for the gE antibody, as expected, both vaccinated pigs and unvaccinated pigs did not develop any gE antibodies before PRV HN1201 challenge, and only vaccinated pigs showed positive gE antibody responses after 10 days of postchallenge (Figure 6(b)). Consistent with gB antibody response, PRV HN1201 specific neutralizing antibodies could be detected after 14 dpv and kept increasing even after PRV HN1201 challenge (Figure 6(c)).

## 4. Discussion

Pseudorabies was once well controlled by vaccinating pigs with gene-deleted modified-live vaccines accompanied by the application of ELISA serologic tests. However, the massive outbreaks of PR happened in vaccinated pigs in China since 2011 and caused huge economic losses to local swine industry [4]. The new-emerging PRV variants are antigenically different from classical PRV and current commercial available PRV vaccines cannot provide complete protection to the circulating PRV field isolates [5, 6]. The origin of current circulating PRV variants still remains unknown, but the massive use of modified-live vaccines could pose the pressure to the evolution of viruses. Therefore it is urgent to develop more effective and safe killed PRV vaccines to control the disease.

The gE gene of PRV is important for virulence and spread of the virus but is dispensable for viral replication [8]. The gE-deleted PRV vaccine has the advantages of differentiating infected from vaccinated animals along with gE ELISA development. Therefore, in this study, we first rescued gE-deleted PRV HN1201ΔgE by using recombination techniques. Compared to the parental PRV HN1201, the rescued HN1201ΔgE virus showed similar growth properties on the PK-15 cells. However, the size of plaques of viruses was much smaller than the parental virus. The size of plaques of some viruses such as influenza virus is related to the virus virulence [9]. Therefore, we test the efficacy of this virus as vaccine candidate on 3-week-old pigs after inactivating virus and combining with commercial adjuvant MONTANIDE ISA 206.

After vaccination, there were no pigs which showed any clinical symptoms including fever which prove the safety of this killed vaccine. The pigs were then challenged with virulent PRV HN1201 to test the efficacy of vaccine. The unvaccinated pigs started to show PR-specific clinical symptoms at 1 dpc and died between 4 and 6 dpc, which showed the high virulence of PRV HN1201 variant. By contrast, the vaccinated pigs did not show any clinical symptoms except transient fever which proved the efficacy of vaccine to PRV HN1201 challenge. We did not include any commercial PRV vaccine immunized pigs in this study since the results from other research groups already proved that current commercial vaccine can only provide limited protection to the PRV variants [10, 11].

Vaccinated pigs developed high level of gB antibodies after vaccination and the antibody titer raised to even higher level after PRV HN1201 challenge (Figure 6(a)). Consistent with gB antibody response, the protective neutralizing antibodies kept increasing even after PRV HN1201 challenge (Figure 6(c)). As expected, the vaccinated pigs did not develop any gE antibody until 10 dpc (Figure 6(b)), and the gE antibodies were generated by the infection of PRV HN1201 in vaccinated pigs since the gE gene was already wiped out from vaccine virus PRV HN1201ΔgE.

Both inactivated and modified-live vaccines based on current circulating PRV variants have been developed [10, 11]. In Gu's study, a gE/gI double gene-deleted killed vaccine can generate high level of gB and neutralizing antibodies and provide complete protection to the PRV variant ZJ01 challenge [11]. There is no difference between deleting two genes (gE/gI) and one gene (gE) to make the DIVA vaccine since currently widely used differentiating ELISA kit is based on the absence of gE gene. However, the gE single gene-deleted PRV vaccine can generate earlier gB antibodies as compared to above-mentioned gE/gI-deleted killed vaccine but reduced neutralizing antibodies in the same window period. In another study, a gE-deleted PRV attenuated live vaccine can also protect the vaccinated pigs from PRV variant challenge and gB-specific antibodies can be detected at 6 dpi [10]. The different window of gB antibody generation could be explained by different types of vaccines (live vaccine versus killed vaccine) and age of pigs used in the study (6-week-old pigs were used in their study). If gB antibodies play a role in the immunity elicited by vaccination, attenuated modified-live vaccine has the advantages as it can generate earlier and higher level of gB antibodies. However, the biosecurity should be taken into consideration since the attenuated live vaccine has the potential to revert virulence. Besides humoral antibody responses, the cellular immunity could also play an important role in elimination of PRV in the tissues and need to be further explored.

PRV antigen cannot be detected in vaccinated pigs after PRV HN1201 challenge by using immunohistochemistry staining. By contrast, there was strong staining in tonsil, lung, lymph node, and cerebellum samples in unvaccinated pigs. Therefore, the immunity developed by vaccination with this killed vaccine can successfully eliminate the challenged virus PRV HN1201 from the pig tissues. Consistent with immunohistochemistry results, the vaccinated pigs did not have any obvious changes in the above tissues by histopathological examination. On the contrary, the unvaccinated pigs showed PR-specific lesions in multiple tissues such as severe hemorrhages and congestion in the lungs and necrosis in the tonsil, lymph node, and cerebellum samples.

Virus shedding works as one of important parameters to evaluate the efficacy of killed PRV vaccine [12]. In this study, there were two vaccinated pigs which excreted PRV at 2 dpc and lasted for 3 days. On the contrary, there were two unvaccinated pigs which excreted virus at 1 dpc and all pigs excreted virus after that until death (Table 2). The lower number of virulent PRV excreted-pigs and shorter period of virus excretion in vaccinated pigs as compared with unvaccinated ones partially showed the efficacy of the vaccine.

In conclusion, we constructed a gE-deleted PRV based on a current circulating field isolate and tested the efficacy of this killed vaccine combined with adjuvant on 3-week-old pigs. The results showed that vaccination can provide complete protection to the ensuing virulent PRV HN1201 challenge. Therefore, this inactivated HN1201ΔgE vaccine could be a promising vaccine candidate for controlling the wide spreading variant strains of PRV in China.

## Figures and Tables

**Table 1:** Sequences of oligonucleotides used in PCR. Underlined nucleotide sequences are corresponding restriction enzyme sites.

Primer	Sequence	Details
TKA-F	5′-CCGgaattcCAAACACCAGCAGGGGCACGA-3′	Left homologous arm of TK gene
TKA-R	5′-CTAGtctagaATAACTTCGTATAATGTATGCTATACGAAGTTATTGTTGACCAGCATGGCGTAGACG-3′	
TKB-F	5′-ACATgcatgcAACGACGACGGCGTGGGAGG-3′	Right homologous arm of TK gene
TKB-R	5′-CCCaagcttAGGGCGACGGCGAAGAAGAGC-3′	
CMVU	5′-ACGCGTCGACTAGTTATTAATAGTAATCAATTACG-3′	
SV40R	5′-GGCCGACGTCGACCTAGAATGCAGTGAAAAAAATGC-3′	
gpt-F	5′-CCGCTCGAGCTATGAGCGAAAAATACATCGTCAC-3′	
gpt-R	5′-CGCGGATCCGCGACCGGAGATTGGCGGGACGA-3′	
PRVgE-F	5′-CTTCCACTCGCAGCTCTTCTC-3′	PCR to detect virus shedding from nasal swabs
PRVgE-R	5′-GTTAAGTTCTCGCGCGAGT-3′	

**Table 2:** The number of pigs excreting virulent PRV from nasal swabs after viral challenge.

Group	0 dpc	1 dpc	2 dpc	3 dpc	4 dpc	5 dpc	6 dpc	7 dpc
Vaccinated	0	0	2	2	1	0	0	0
Unvaccinated	0	2	5	5	5	1	1	N/A

N/A = not applicable.
